# 5,5′-Dichloro-2,2′-dimeth­oxy­biphen­yl

**DOI:** 10.1107/S160053681300826X

**Published:** 2013-04-05

**Authors:** Hans-Joachim Lehmler, Huimin Wu, Sean Parkin

**Affiliations:** a The University of Iowa, Department of Occupational and Environmental Health, Iowa City, IA 52242-5000, USA; b University of Kentucky, Department of Chemistry, Lexington, KY 40506-0055, USA

## Abstract

In the title mol­ecule, C_14_H_12_Cl_2_O_2_, the dihedral angle between the least-square planes of the benzene rings is 62.17 (6)°. Both meth­oxy groups are slightly out of the plane of the benzene rings to which they are attached, making dihedral angles of 4.22 (18) and 18.82 (16)°.

## Related literature

For background to polychlorinated biphenyls, see: Basu *et al.* (2009 ▶); Hu *et al.* (2008 ▶); Kaminsky *et al.* (1981 ▶); Kennedy *et al.* (1981 ▶); McLean *et al.* (1996 ▶); Rodenburg *et al.* (2010 ▶). For related structures, see: Chattopadhyay *et al.* (1987 ▶); Nakaema *et al.* (2008 ▶); Sun *et al.* (2001 ▶). For the synthesis of the title compound, see: Joshi *et al.* (2011 ▶).
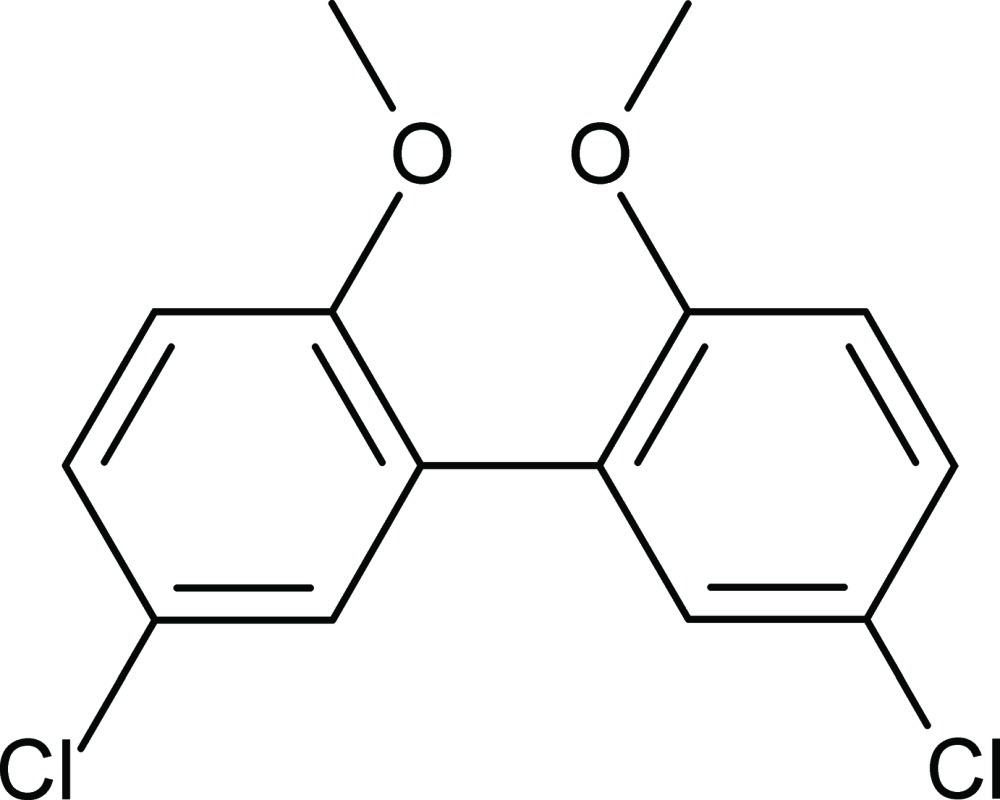


## Experimental

### 

#### Crystal data

C_14_H_12_Cl_2_O_2_
*M* *_r_* = 283.14
Monoclinic, 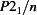

*a* = 10.9629 (2) Å
*b* = 7.2177 (1) Å
*c* = 16.7812 (3) Å
β = 104.7108 (7)°
*V* = 1284.32 (4) Å^3^
*Z* = 4
Mo *K*α radiation
μ = 0.50 mm^−1^
*T* = 90 K
0.22 × 0.20 × 0.18 mm

#### Data collection

Nonius KappaCCD diffractometer
Absorption correction: multi-scan (*SCALEPACK*; Otwinowski & Minor, 1997 ▶) *T*_min_ = 0.899, *T*_max_ = 0.916
21357 measured reflections
2948 independent reflections
2347 reflections with *I* > 2σ(*I*)
*R*_int_ = 0.047

#### Refinement

*R*[*F* ^2^ > 2σ(*F* ^2^)] = 0.042
*wR*(*F* ^2^) = 0.111
*S* = 1.11
2948 reflections
165 parameters
H-atom parameters constrained
Δρ_max_ = 0.43 e Å^−3^
Δρ_min_ = −0.41 e Å^−3^

### 

Data collection: *COLLECT* (Nonius, 1998 ▶); cell refinement: *SCALEPACK* (Otwinowski & Minor, 1997 ▶); data reduction: *DENZO-SMN* (Otwinowski & Minor, 1997 ▶); program(s) used to solve structure: *SHELXS97* (Sheldrick, 2008 ▶); program(s) used to refine structure: *SHELXL97* (Sheldrick, 2008 ▶); molecular graphics: *XP* in *SHELXTL* (Sheldrick, 2008 ▶); software used to prepare material for publication: *SHELXL97* and local procedures.

## Supplementary Material

Crystal structure: contains datablock(s) I, global. DOI: 10.1107/S160053681300826X/lh5598sup1.cif

Structure factors: contains datablock(s) I. DOI: 10.1107/S160053681300826X/lh5598Isup2.hkl

Supplementary material file. DOI: 10.1107/S160053681300826X/lh5598Isup3.cml

Additional supplementary materials:  crystallographic information; 3D view; checkCIF report

## Figures and Tables

**Fig. 1.**
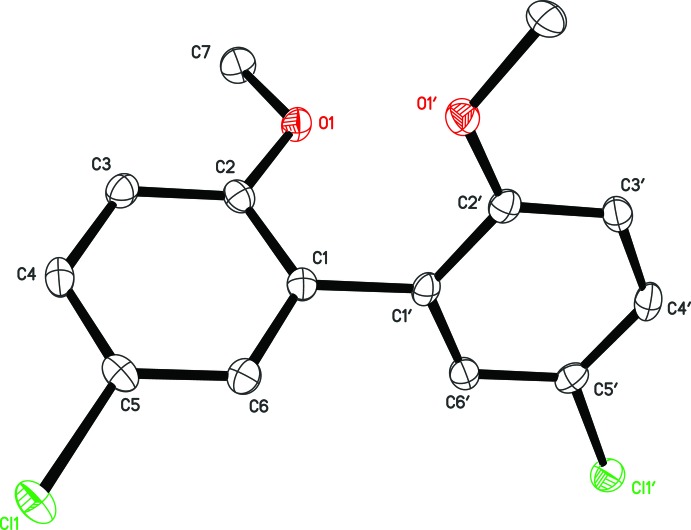
View of the title compound showing displacement ellipsoids drawn at the 50% probability level.

## supplementary crystallographic information

### Comment

3,3'-Dichlorobiphenyl (CB11) is a polychlorinated biphenyl (PCB) congener found at comparatively high concentrations in water and air (Basu *et al.*, 2009; Hu *et al.*, 2008; Rodenburg *et al.*, 2010). It is probably produced during the manufacturing of paint pigments and/or the degradation of higher chlorinated PCBs (Basu *et al.*, 2009). Early metabolism studies with CB11 demonstrate its oxidation to mono-hydroxylated metabolites by hepatic cytochrome P450 enzymes (Kaminsky *et al.*, 1981; Kennedy *et al.*, 1981). Similar to other lower chlorinated PCB congeners (McLean *et al.*, 1996), it is likely that these mono-hydroxylated metabolites are further oxidized to dihydroxylated metabolites, such as 2,2'-dihydroxy PCB 11. Here we report the crystal structure of the title compound, a dimethylated derivative of 2,2'-dihydroxy PCB 11.

There is one molecule of the title compound in the asymmetric unit (Fig. 1). The dihedral angle between the least-square planes of the two aromatic rings is 62.17 (6)°. The corresponding dihedral angles of structurally related biphenyls, 2,2'-dimethoxybiphenyls, 4,4'-dimethyl-2,2',5,5'-dimethoxybiphenyl and 2,2',4,4',5,5'-hexamethoxybiphenyl, are comparable with 66.94 (7)°, 69.1° (no s.u. available) and 81.1 (1)°, respectively (Nakaema *et al.*, 2008; Sun *et al.*, 2001; Chattopadhyay *et al.*, 1987). In the title compound, the methoxy group at C7' has an almost coplanar arrangement with aromatic ring system, with a dihedral angle of 4.22 (18)°. In contrast, the methoxy group at C7, with a dihedral angle of 18.82 (16)°, is slightly out of the plane of the aromatic ring system. The twist of this methoxy group out of the plane of the aromatic ring system is relatively large compared to related crystal structures, which display dihedral angles ranging from 1.0° to 12.2° (Nakaema *et al.*, 2008; Sun *et al.*, 2001; Chattopadhyay *et al.*, 1987).

### Experimental

The title compound was prepared by the Suzuki coupling of 2-iodo-4-chloroanisole and 2-methoxy-5-chlorobenzeneboronic acid as described previously (Joshi *et al.*, 2011). Crystals suitable for crystal structure analysis were obtained by slow evaporation of a methanolic solution.

### Refinement

H atoms were found in difference Fourier maps and subsequently placed in idealized positions with constrained distances of 0.98 Å (RCH_3_), 0.95 Å (C_Ar_H), and with *U*_iso_(H) values set to either 1.2*U*_eq_ or 1.5*U*_eq_ (RCH_3_) of the attached atom.

### Crystal data

**Table:**

C_14_H_12_Cl_2_O_2_	*F*(000) = 584
*M**_r_* = 283.14	*D*_x_ = 1.464 Mg m^−^^3^
Monoclinic, *P*2_1_/*n*	Mo *K*α radiation, λ = 0.71073 Å
Hall symbol: -P 2yn	Cell parameters from 3160 reflections
*a* = 10.9629 (2) Å	θ = 1.0–27.5°
*b* = 7.2177 (1) Å	µ = 0.50 mm^−^^1^
*c* = 16.7812 (3) Å	*T* = 90 K
β = 104.7108 (7)°	Block, colourless
*V* = 1284.32 (4) Å^3^	0.22 × 0.20 × 0.18 mm
*Z* = 4	

### Data collection

**Table:**

Nonius KappaCCD diffractometer	2948 independent reflections
Radiation source: fine-focus sealed tube	2347 reflections with *I* > 2σ(*I*)
Graphite monochromator	*R*_int_ = 0.047
Detector resolution: 9.1 pixels mm^-1^	θ_max_ = 27.5°, θ_min_ = 2.0°
ω scans at fixed χ = 55°	*h* = −14→14
Absorption correction: multi-scan (*SCALEPACK*; Otwinowski & Minor, 1997)	*k* = −9→9
*T*_min_ = 0.899, *T*_max_ = 0.916	*l* = −21→21
21357 measured reflections	

### Refinement

**Table:**

Refinement on *F*^2^	Primary atom site location: structure-invariant direct methods
Least-squares matrix: full	Secondary atom site location: difference Fourier map
*R*[*F*^2^ > 2σ(*F*^2^)] = 0.042	Hydrogen site location: inferred from neighbouring sites
*wR*(*F*^2^) = 0.111	H-atom parameters constrained
*S* = 1.11	*w* = 1/[σ^2^(*F*_o_^2^) + (0.0529*P*)^2^ + 0.9091*P*] where *P* = (*F*_o_^2^ + 2*F*_c_^2^)/3
2948 reflections	(Δ/σ)_max_ < 0.001
165 parameters	Δρ_max_ = 0.43 e Å^−^^3^
0 restraints	Δρ_min_ = −0.41 e Å^−^^3^

### Special details

**Table:**

Geometry. All e.s.d.'s (except the e.s.d. in the dihedral angle between two l.s. planes) are estimated using the full covariance matrix. The cell e.s.d.'s are taken into account individually in the estimation of e.s.d.'s in distances, angles and torsion angles; correlations between e.s.d.'s in cell parameters are only used when they are defined by crystal symmetry. An approximate (isotropic) treatment of cell e.s.d.'s is used for estimating e.s.d.'s involving l.s. planes.
Refinement. Refinement of *F*^2^ against all reflections. The weighted *R*-value *wR* and goodness of fit *S* are based on *F*^2^. Conventional *R*-values *R* are based on *F*, with *F* set to zero for negative *F*^2^. The threshold expression of *F*^2^ > 2σ(*F*^2^) is used only for calculating *R*-factors(gt) *etc*. and is not relevant to the choice of reflections for refinement. *R*-values based on *F*^2^ are statistically about twice as large as those based on *F*, and *R*-values based on ALL data will be even larger.

### Fractional atomic coordinates and isotropic or equivalent isotropic displacement parameters (Å^2^)

**Table:**

	*x*	*y*	*z*	*U*_iso_*/*U*_eq_	
Cl1	0.07535 (4)	0.15602 (7)	0.32405 (3)	0.02285 (15)	
O1	0.56298 (12)	0.53852 (19)	0.38856 (9)	0.0168 (3)	
C1	0.43664 (17)	0.2807 (3)	0.33719 (12)	0.0137 (4)	
C2	0.45037 (18)	0.4500 (3)	0.37970 (12)	0.0149 (4)	
C3	0.35230 (18)	0.5203 (3)	0.40971 (12)	0.0170 (4)	
H3	0.3636	0.6321	0.4406	0.020*	
C4	0.23774 (18)	0.4264 (3)	0.39443 (12)	0.0171 (4)	
H4	0.1709	0.4726	0.4154	0.020*	
C5	0.22250 (18)	0.2655 (3)	0.34850 (12)	0.0170 (4)	
C6	0.32045 (18)	0.1903 (3)	0.32061 (12)	0.0163 (4)	
H6	0.3085	0.0776	0.2903	0.020*	
C7	0.56729 (19)	0.7317 (3)	0.40866 (14)	0.0211 (4)	
H7A	0.4958	0.7952	0.3718	0.032*	
H7B	0.6463	0.7851	0.4020	0.032*	
H7C	0.5628	0.7470	0.4659	0.032*	
Cl1'	0.59609 (5)	0.10229 (8)	0.08207 (3)	0.02197 (15)	
O1'	0.66596 (13)	0.1598 (2)	0.44014 (8)	0.0174 (3)	
C1'	0.54159 (18)	0.2061 (3)	0.30496 (12)	0.0137 (4)	
C2'	0.65747 (18)	0.1509 (3)	0.35747 (12)	0.0147 (4)	
C3'	0.75356 (18)	0.0855 (3)	0.32448 (12)	0.0163 (4)	
H3'	0.8319	0.0501	0.3603	0.020*	
C4'	0.73626 (19)	0.0711 (3)	0.23964 (13)	0.0175 (4)	
H4'	0.8024	0.0277	0.2173	0.021*	
C5'	0.62136 (18)	0.1210 (3)	0.18870 (12)	0.0157 (4)	
C6'	0.52457 (18)	0.1888 (3)	0.22031 (12)	0.0156 (4)	
H6'	0.4465	0.2235	0.1839	0.019*	
C7'	0.78413 (19)	0.1104 (3)	0.49485 (13)	0.0212 (5)	
H7'1	0.8051	−0.0177	0.4841	0.032*	
H7'2	0.7782	0.1214	0.5520	0.032*	
H7'3	0.8501	0.1935	0.4860	0.032*	

### Atomic displacement parameters (Å^2^)

**Table:**

	*U*^11^	*U*^22^	*U*^33^	*U*^12^	*U*^13^	*U*^23^
Cl1	0.0134 (2)	0.0267 (3)	0.0286 (3)	−0.00368 (19)	0.0056 (2)	0.0006 (2)
O1	0.0159 (7)	0.0120 (7)	0.0239 (8)	−0.0027 (5)	0.0075 (6)	−0.0041 (6)
C1	0.0140 (9)	0.0144 (9)	0.0130 (9)	0.0016 (7)	0.0040 (7)	0.0015 (8)
C2	0.0135 (9)	0.0158 (10)	0.0151 (9)	0.0010 (8)	0.0032 (7)	0.0029 (8)
C3	0.0188 (10)	0.0146 (10)	0.0178 (10)	0.0016 (8)	0.0048 (8)	−0.0016 (8)
C4	0.0146 (9)	0.0195 (10)	0.0186 (10)	0.0038 (8)	0.0070 (8)	0.0012 (8)
C5	0.0127 (9)	0.0195 (10)	0.0184 (10)	−0.0010 (8)	0.0031 (7)	0.0035 (8)
C6	0.0172 (10)	0.0152 (10)	0.0167 (10)	0.0009 (8)	0.0048 (8)	−0.0001 (8)
C7	0.0218 (10)	0.0122 (10)	0.0293 (12)	−0.0031 (8)	0.0065 (9)	−0.0035 (9)
Cl1'	0.0204 (3)	0.0295 (3)	0.0170 (3)	−0.0005 (2)	0.00670 (19)	−0.0048 (2)
O1'	0.0169 (7)	0.0198 (7)	0.0153 (7)	0.0038 (6)	0.0036 (5)	0.0010 (6)
C1'	0.0153 (9)	0.0084 (9)	0.0189 (10)	−0.0007 (7)	0.0069 (7)	−0.0015 (8)
C2'	0.0165 (9)	0.0113 (9)	0.0172 (10)	−0.0017 (7)	0.0058 (8)	−0.0009 (8)
C3'	0.0135 (9)	0.0140 (9)	0.0215 (10)	0.0008 (8)	0.0046 (8)	−0.0002 (8)
C4'	0.0169 (9)	0.0135 (10)	0.0250 (11)	0.0007 (8)	0.0109 (8)	−0.0015 (8)
C5'	0.0197 (10)	0.0146 (10)	0.0142 (9)	−0.0031 (8)	0.0069 (8)	−0.0020 (8)
C6'	0.0142 (9)	0.0127 (9)	0.0201 (10)	−0.0004 (7)	0.0047 (8)	0.0000 (8)
C7'	0.0202 (10)	0.0224 (11)	0.0177 (10)	0.0052 (9)	−0.0016 (8)	0.0004 (9)

### Geometric parameters (Å, º)

**Table:**

Cl1—C5	1.749 (2)	Cl1'—C5'	1.745 (2)
O1—C2	1.364 (2)	O1'—C2'	1.368 (2)
O1—C7	1.432 (2)	O1'—C7'	1.430 (2)
C1—C6	1.395 (3)	C1'—C6'	1.391 (3)
C1—C2	1.404 (3)	C1'—C2'	1.407 (3)
C1—C1'	1.491 (2)	C2'—C3'	1.391 (3)
C2—C3	1.393 (3)	C3'—C4'	1.392 (3)
C3—C4	1.392 (3)	C3'—H3'	0.9500
C3—H3	0.9500	C4'—C5'	1.379 (3)
C4—C5	1.381 (3)	C4'—H4'	0.9500
C4—H4	0.9500	C5'—C6'	1.390 (3)
C5—C6	1.386 (3)	C6'—H6'	0.9500
C6—H6	0.9500	C7'—H7'1	0.9800
C7—H7A	0.9800	C7'—H7'2	0.9800
C7—H7B	0.9800	C7'—H7'3	0.9800
C7—H7C	0.9800		
			
C2—O1—C7	117.14 (15)	C2'—O1'—C7'	117.18 (15)
C6—C1—C2	118.90 (17)	C6'—C1'—C2'	118.68 (17)
C6—C1—C1'	120.57 (17)	C6'—C1'—C1	119.16 (17)
C2—C1—C1'	120.38 (16)	C2'—C1'—C1	122.16 (17)
O1—C2—C3	123.39 (18)	O1'—C2'—C3'	123.77 (18)
O1—C2—C1	116.12 (16)	O1'—C2'—C1'	116.10 (16)
C3—C2—C1	120.48 (17)	C3'—C2'—C1'	120.09 (18)
C4—C3—C2	119.96 (19)	C2'—C3'—C4'	120.81 (18)
C4—C3—H3	120.0	C2'—C3'—H3'	119.6
C2—C3—H3	120.0	C4'—C3'—H3'	119.6
C5—C4—C3	119.19 (18)	C5'—C4'—C3'	118.72 (18)
C5—C4—H4	120.4	C5'—C4'—H4'	120.6
C3—C4—H4	120.4	C3'—C4'—H4'	120.6
C4—C5—C6	121.58 (18)	C4'—C5'—C6'	121.41 (18)
C4—C5—Cl1	119.00 (15)	C4'—C5'—Cl1'	119.85 (15)
C6—C5—Cl1	119.41 (16)	C6'—C5'—Cl1'	118.74 (15)
C5—C6—C1	119.72 (18)	C5'—C6'—C1'	120.26 (18)
C5—C6—H6	120.1	C5'—C6'—H6'	119.9
C1—C6—H6	120.1	C1'—C6'—H6'	119.9
O1—C7—H7A	109.5	O1'—C7'—H7'1	109.5
O1—C7—H7B	109.5	O1'—C7'—H7'2	109.5
H7A—C7—H7B	109.5	H7'1—C7'—H7'2	109.5
O1—C7—H7C	109.5	O1'—C7'—H7'3	109.5
H7A—C7—H7C	109.5	H7'1—C7'—H7'3	109.5
H7B—C7—H7C	109.5	H7'2—C7'—H7'3	109.5
			
C7—O1—C2—C3	−17.7 (3)	C6—C1—C1'—C2'	−119.2 (2)
C7—O1—C2—C1	161.82 (17)	C2—C1—C1'—C2'	65.4 (3)
C6—C1—C2—O1	−175.20 (17)	C7'—O1'—C2'—C3'	4.5 (3)
C1'—C1—C2—O1	0.3 (3)	C7'—O1'—C2'—C1'	−177.96 (17)
C6—C1—C2—C3	4.3 (3)	C6'—C1'—C2'—O1'	−175.93 (17)
C1'—C1—C2—C3	179.85 (18)	C1—C1'—C2'—O1'	3.6 (3)
O1—C2—C3—C4	176.63 (18)	C6'—C1'—C2'—C3'	1.7 (3)
C1—C2—C3—C4	−2.9 (3)	C1—C1'—C2'—C3'	−178.75 (18)
C2—C3—C4—C5	−0.9 (3)	O1'—C2'—C3'—C4'	176.60 (18)
C3—C4—C5—C6	3.2 (3)	C1'—C2'—C3'—C4'	−0.9 (3)
C3—C4—C5—Cl1	−176.05 (15)	C2'—C3'—C4'—C5'	−0.7 (3)
C4—C5—C6—C1	−1.7 (3)	C3'—C4'—C5'—C6'	1.5 (3)
Cl1—C5—C6—C1	177.55 (15)	C3'—C4'—C5'—Cl1'	−179.32 (15)
C2—C1—C6—C5	−2.1 (3)	C4'—C5'—C6'—C1'	−0.6 (3)
C1'—C1—C6—C5	−177.59 (18)	Cl1'—C5'—C6'—C1'	−179.82 (15)
C6—C1—C1'—C6'	60.3 (3)	C2'—C1'—C6'—C5'	−1.0 (3)
C2—C1—C1'—C6'	−115.1 (2)	C1—C1'—C6'—C5'	179.49 (17)
